# Low-intensity continuous ultrasound triggers effective bisphosphonate anticancer activity in breast cancer

**DOI:** 10.1038/srep16354

**Published:** 2015-11-18

**Authors:** Sophie Tardoski, Jacqueline Ngo, Evelyne Gineyts, Jean-Paul Roux, Philippe Clézardin, David Melodelima

**Affiliations:** 1 Inserm, U1032, Lyon, F-69424, France; 2 Inserm, U1033, Lyon, F-69372, France; 3 University of Lyon, Villeurbanne, France; 4 LabEx DEVweCAN, Lyon, France

## Abstract

Ultrasound (US) is a non-ionizing pressure wave that can produce mechanical and thermal effects. Bisphosphonates have demonstrated clinical utility in bone metastases treatment. Preclinical studies suggest that bisphosphonates have anticancer activity. However, bisphosphonates exhibit a high affinity for bone mineral, which reduces their bioavailibity for tumor cells. Ultrasound has been shown to be effective for drug delivery but in interaction with gas bubbles or encapsulated drugs. We examined the effects of a clinically relevant dose of bisphosphonate zoledronate (ZOL) in combination with US. In a bone metastasis model, mice treated with ZOL+US had osteolytic lesions that were 58% smaller than those of ZOL-treated animals as well as a reduced skeletal tumor burden. In a model of primary tumors, ZOL+US treatment reduced by 42% the tumor volume, compared with ZOL-treated animals. Using a fluorescent bisphosphonate, we demonstrated that US forced the release of bisphosphonate from the bone surface, enabling a continuous impregnation of the bone marrow. Additionally, US forced the penetration of ZOL within tumors, as demonstrated by the intratumoral accumulation of unprenylated Rap1A, a surrogate marker of ZOL antitumor activity. Our findings made US a promising modality to trigger bisphosphonate anticancer activity in bone metastases and in primary tumors.

Breast cancer is prone to metastasize to bone: around 80% of patients with advanced disease exhibit osteolytic bone metastases^1^,^2^. Once metastatic breast cells are in the bone marrow, they alter the functions of bone-resorbing (osteoclasts) and bone-forming (osteoblasts) cells and hijack signals coming from the bone matrix^1^,^2^. Specifically, metastatic breast cancer cells enhance bone resorption and inhibit bone formation, which leads to skeletal destruction and subsequent occurrence of skeletal complications^1^. These skeletal complications can be fatal or may rapidly impede the quality of life of patients by causing pathological fractures, hypercalcemia, nerve compression and loss of mobility^2^.

Bisphosphonates are bone-seeking drugs that inhibit osteoclast-mediated bone resorption^2^. They are the standard of care for the prevention and treatment of skeletal complications in patients with malignant bone disease^2^. Additionally, a body of preclinical evidence suggests that bisphosphonates may act as antitumor agents, able to inhibit tumor cell adhesion, invasion and proliferation *in vitro*^3^. Bisphosphonates also inhibit tumor growth *in vivo* through antiangiogenic, anti-invasive and immunomodulatory activities^3^,^4^,^5^. However, bisphosphonates bind avidly to bone mineral, which limits their bioavailability and therefore their direct antitumor potential *in vivo*^2^,^6^. This observation likely explains why high doses of bisphosphonates have often been used in experimental studies. Unfortunately, due to renal toxicity, such high doses are incompatible with the bisphosphonate dosing regimens that have been approved for the treatment of patients with bone metastases^2^.

Current advances in acoustic technology have made ultrasound a modality with therapeutic and diagnostic applications in oncology^7^,^8^. Ultrasound energy is a non-ionizing pressure wave that can produce both mechanical and thermal effects^8^. In this respect, high-intensity focused ultrasound has potential for tumor ablation, causing tissue necrosis through the conversion of mechanical energy into heat (up to 80–90 °C within tissues) and inertial cavitation (that is formation and immediate and violent collapse of gas-filled bubbles)^7^,^9^,^10^. Mild temperatures (around 43 °C) called hyperthermia can also be created using ultrasound. Hyperthermia leads to increased blood flow and enhanced vascular permeability and tumor oxygenation, promoting effectiveness of chemotherapy^11^. Ultrasound can also enhance local delivery of drugs or genes through stable cavitation (that is the sustained growth of cavitation bubbles and their oscillations over several acoustic cycles) and mostly inertial cavitation^12^. Stable cavitation is thought to enhance permeability of blood vessels by creating gaps between endothelial cells^12^. Ultrasound can also initiate the release of drugs from liposomes via inertial cavitation, which – through the rarefactional phase of an ultrasound wave - causes the expansion of a gas bubble followed by a violent collapse due to the inertia of the surrounding media. This collapse creates shock waves, which can disrupt the stability of co-localized liposomal drug carriers^13^. However, producing cavitation for drug/gene delivery without damaging tissues may be difficult to predict and control *in vivo*^14^,^15^. Interestingly, when used at a low intensity, pulsed ultrasound can enhance bone fracture repair by inducing a mechanical stress in bone, which in turn, stimulate the ossification of a soft callus through the modulation of calcium ions channel^16^,^17^,^18^,^19^. Because bone destruction not only results from enhanced osteoclast-mediated bone resorption but also of inhibition of bone formation^1^, low-intensity ultrasound could be therefore an effective strategy in the treatment of bone metastasis to restore osteoblast functions^20^. The exact mechanisms leading to bone growth stimulated by low intensity pulsed ultrasound still need to be more thoroughly understood. Low-intensity ultrasound applied continuously might even enhance local delivery of bisphosphonates in skeletal tumors by creating mild hyperthermia and biomechanical effects, while avoiding cavitation.

Here, we sought to explore the effects of low-intensity continuous ultrasound as a strategy to force bisphosphonate penetration through tumor tissue using animal models of human breast cancer and bone metastasis. Results demonstrated significantly reduced bone destruction and slower tumor burden, both in bone and outside bone, when using ultrasound in combination with the bisphosphonate zoledronate.

## Results

### Mild hyperthermia (HT) enhances antitumor activity of zoledronate (ZOL) *in vitro*

Isopentenyl pyrophosphate (IPP) accumulation is commonly used as a surrogate marker of bisphosphonate penetration in tumor cells^21^. Cells treated with PBS (vehicle) or HT alone did not accumulate IPP (Fig. S1A). No IPP accumulation was observed with a 1-hour treatment of ZOL, combined or not with HT (Fig. S1A). Accumulation of IPP in tumor cells was observed with a 24-hours treatment of ZOL (1054 ± 310 pmol/mg). If a 24-hours treatment of ZOL was combined with 30 minutes of HT a significant increase in the intracellular accumulation of IPP was observed when compared with ZOL alone (2002 ± 235 pmol/mg; p < 0.05) (Fig. S1A).

### Low intensity ultrasound (US) enhances antitumor activity of zoledronate (ZOL) *in vitro*

The bisphosphonate ZOL is a potent inhibitor of farnesyl pyrophosphate (FPP) synthase, a key enzyme in the mevalonate pathway^3^. As a result of the inhibition of FPP synthase, ZOL prevents the prenylation of small G-proteins (*e.g*., Ras, Rho and Rap1A), which are essential for cancer cell adhesion, migration and invasion, as it induces the intracellular accumulation of the ATP analogue isopentenyl pyrophosphate (IPP) in tumor cells^3^. Measurements of unprenylated small G-proteins (*i.e*., Rap1A) and IPP in ZOL-treated tumor cells have been therefore used as surrogate markers of bisphosphonate antitumor activity^3^.

**Figure 1:**
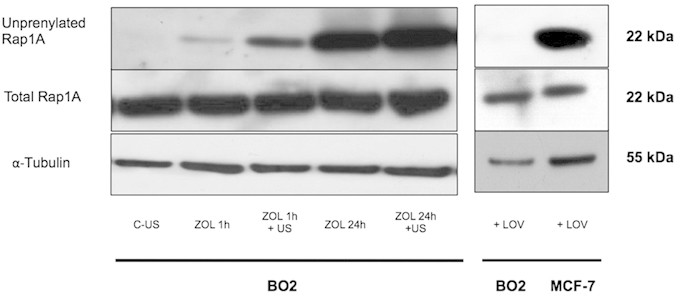
Low-intensity continuous ultrasound (US) enhances the antitumor effect of the bisphosphonate zoledronate (ZOL) *in vitro*. B02 breast cancer cells were treated with 25-μM ZOL for 1h, then cultured without drug for 23h. C-US was given for 30 min. B02 cells in culture also received a 24-h ZOL treatment, alone or in combination with C-US (30 min). Alternatively, B02 and MCF-7 breast cancer cells were treated with 5 μM of the HMG-CoA reductase inhibitor lovastatin (LOV). Cells were harvested, lysed and protein extracts were electrophoresed then analyzed by western blotting for the presence of unprenylated and total Rap1A. Cropped gels are presented. Full-length blots are presented in Supplementary Figure 3. Tubulin was used as a control for equal protein loading. LOV-treated MCF-7 cells were used as a positive control.

The effects of ZOL and US on IPP accumulation in human MDA-MB-231/B02 breast cancer cells *in vitro* was examined by mass spectrometry (Fig. S1). B02 cells treated with PBS (the vehicle for ZOL) or US alone did not accumulate IPP. By contrast, a one-hour ZOL treatment in combination with US increased statistically significantly the intracellular accumulation of IPP in tumor cells, compared with either treatment alone (1,967 ± 882 and 109 ± 61 pmol/mg protein in US+ZOL- and ZOL-treated tumor cells, respectively; *P* < 0.01). A 24-hours ZOL treatment in combination with US further increased intracellular IPP levels in tumor cells, compared with ZOL alone (7,500 ± 657 vs 3,200 ± 703 pmol/mg; *P* < 0.01) (Fig. S1). Combining US with ZOL allowed the highest accumulation of IPP compared to all other groups, including pure hyperthermia treatments (p < 0.05) (Fig. S1). Additionally, we found a strong correlation between IPP accumulation and unprenylation of Rap1A in B02 cells treated with ZOL, alone or in combination with US (Fig. 1). As shown by Western blotting, unprenylated Rap1A was undetectable in tumor cells treated only with US. Unprenylated Rap1A was barely detectable following a 1-h ZOL treatment of tumor cells. By contrast, when ZOL and US were combined to treat B02 cells for 1 hour a substantially enhanced accumulation of unprenylated Rap1A was observed (Fig. 1). A 24-h treatment with ZOL further increased accumulation of unprenylated Rap1A, irrespective of US (Fig. 1). However, given the short half-life of bisphosphonates in blood (approximately 1 hour), it is unlikely that cancer cells are exposed to a bisphosphonate for several hours *in vivo*.

We previously reported that the activity of the mevalonate pathway in B02 cells was low compared with that of MCF-7 breast cancer cells^4^,^5^. As illustrated in Fig. 1, lovastatin (an inhibitor of HMG-CoA reductase, a mevalonate pathway enzyme upstream of FPP synthase) substantially increased unprenylated Rap1A protein levels in MCF-7 cells. In sharp contrast, unprenylated Rap1A was undetectable in lovastatin-treated B02 cells. Nevertheless, the combined treatment of B02 cells with ZOL+US did induce intracellular accumulation of unprenylated Rap1A in these tumor cells, further demonstrating that US maximized the antitumor effect of ZOL in B02 breast cancer cells *in vitro*.

### Low-intensity continuous ultrasound (US) induced hyperthermia and mechanical stress, while avoiding acoustic cavitation

Measurements performed with an hydrophone demonstrated that wideband emission, which is considered to be a signature for cavitation^22^, was not detected in cultured B02 cells treated for 30 min with US (Fig. S4A), indicating that cavitation was not involved during sonication experiments. By contrast, a 30-min treatment of B02 cells with US induced mild hyperthermia (Fig. S4B). US-induced hyperthermia did not however affect B02 tumor growth at 24-h post-treatment, when used alone or in combination with ZOL (Fig. S5). Similar results were obtained with human MCF-7 or T47D breast cancer cells (Fig. S5).

As opposed to what was observed with ZOL+US treatment (Fig. S1A), a 1-h treatment of B02 cells with ZOL under hyperthermic culture conditions (42 °C) created with warm water did not induce intracellular accumulation of IPP (Fig. S1B and S1C). Similarly, Rap1A was barely detectable under these experimental conditions (Fig. S1C). Thus, these results indicated that US not only induced hyperthermia but also mechanical stress, leading to drug uptake by tumor cells.

We next examined whether US generated similar bioeffects (hyperthermia and/or cavitation) *in vivo*. The experimental device used to treat mice with US is depicted in Fig. S2C, the ultrasounds probe being focused on hind limbs of animals. US treatment of mice induced a mild hyperthermia in the hind limbs, whereas the temperature in the abdominal cavity remained unchanged (Fig. S4D). Examination of main organs (kidneys, liver, spleen, lungs, ovaries), at autopsy and then histologically, revealed that US treatment did not cause any damage (data not shown). Additionally, US treatment did not induce acoustic cavitation in animals (Fig. S4E).

### Low-intensity ultrasound (US) enhances the inhibitory effect of zoledronate (ZOL) on progression of established breast cancer bone metastases

We used a mouse model of human B02 breast cancer bone metastasis in which animals display radiographic evidence of osteolytic lesions in hind limbs 18 days after tumor cell inoculation^23^,^24^. We compared the effects of a single administration of ZOL (100 μg/kg body weight), alone or in combination with US (applied single or daily), on the progression of established bone metastases by using a protocol in which treatment (ZOL and/or US) was administered to tumor-bearing mice on day 18 after tumor cell inoculation. The dosing regimen of ZOL used in the present study was equivalent to the 4-mg clinical intravenous dose given to breast cancer patients with bone metastases.

**Figure 2:**
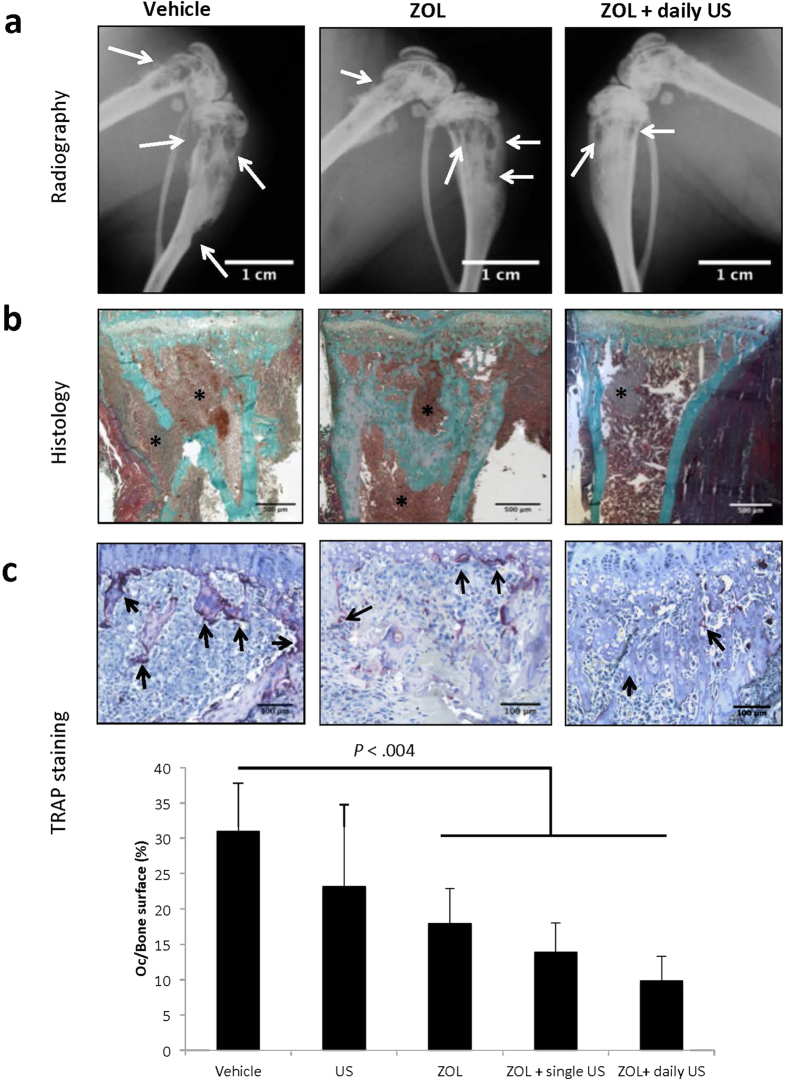
Effects of zoledronate (ZOL), alone or in combination with low-intensity continuous ultrasound (US), on the progression of established breast cancer bone metastases. (**a**) Radiographic analysis of hind limbs from B02 tumor-bearing mice treated with the vehicle, ZOL or the combination of ZOL with US. Arrows indicate osteolytic lesions. (**b**) Goldner’s trichrome staining of tissue sections of tibial metaphysis from metastatic legs. Bone is stained green whereas bone marrow and tumor cells (asterisk) are stained brown. (**c**) *upper panels*: tartrate-resistant acid phosphatase (TRAP) staining of bone tissue sections of metastatic legs from mice, showing osteoclast resorption surfaces (arrows). *Bottom graph:* Osteoclast resorption surface was calculated as the ratio of TRAP-positive trabecular bone surface to the total trabecular bone surface at the tumor-bone interface. All images were obtained from different mice on day 32 after tumor cell inoculation. The images shown are examples that best illustrate the effects of the different treatments on bone metastasis formation.

Radiographic analysis on day 32 after tumor cell injection revealed that tumor-bearing mice treated with a single dose of ZOL had osteolytic lesions that were 55% (*P* < 0.01) smaller than those of tumor-bearing mice treated with the vehicle (Fig. 2a and Table 1). Compared with vehicle, a daily US treatment of tumor-bearing animals did not inhibit bone destruction (Table 1). Similarly, tumor-bearing mice treated with a single injection of ZOL immediately followed by single US treatment had osteolytic lesions that were the same size as those of mice treated with ZOL only (Table 1). In sharp contrast, we found that tumor-bearing mice treated with a single injection of ZOL and receiving a daily treatment with US had osteolytic lesions that were 81% (*P* < 0.001) and 58% (*P* < 0.01) smaller than those of vehicle-treated and ZOL-treated animals, respectively (Table 1 and Fig. 2a).

Histomorphometric analysis of hind limbs with metastases showed that mice treated with ZOL had a statistically significant higher BV/TV ratio (bone volume/tissue volume; indicating prevention of bone loss) than vehicle-treated mice (Fig. 2b and Table 2). This difference was accompanied with a sharp reduction of the tartrate-resistant acid phosphatase (TRAP) staining of bone tissue sections of metastatic legs from ZOL-treated mice (indicating a reduction of active-osteoclast resorption surfaces at the tumor-bone interface) (Fig. 2c). By contrast, the BV/TV ratio and TRAP staining of hind limbs from animals treated with a daily application of US were similar to those observed with vehicle-treated animals (Table 2 and Fig. 2c). The use of US (single or daily application) in combination with ZOL did not further improve the BV/TV ratio, compared with the ZOL-treated group (Table 2). Similarly, a daily application of US in combination with ZOL did not statistically significantly further reduce TRAP staining, compared with that observed with ZOL alone (Fig. 2c). Importantly, the combined treatment of ZOL+US decreased the TB/STV ratio (a measure of the skeletal tumor burden) by 76% (*P* < 0.01), compared with vehicle or US alone (Fig. 2b and Table 2). Additionally, a treatment with ZOL+US was statistically significantly more effective than a treatment with ZOL alone (*P* < 0.01) at decreasing skeletal tumor burden (Fig. 2b and Table 2). Thus, in addition to its therapeutic activity in preserving bone tissue, our histomorphometric data showed that ZOL had anticancer benefits in the treatment of experimental bone metastasis, when used in combination with US.

### Zoledronate (ZOL) in combination with low-intensity ultrasound (US) inhibits tumor-associated angiogenesis and tumor cell proliferation in experimental breast cancer bone metastases

**Figure 3:**
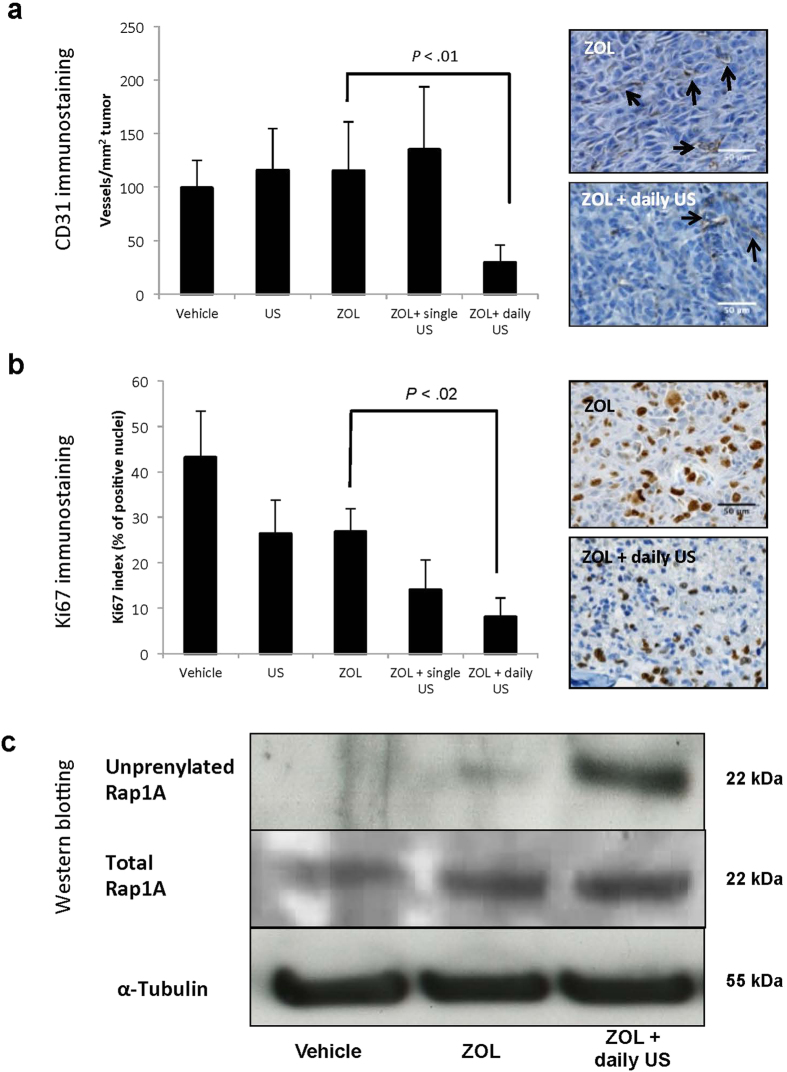
Effects of zoledronate (ZOL), alone or in combination with low-intensity continuous ultrasound (US), on B02 skeletal tumor burden. (**a**) Quantification of CD31-positive blood vessels within skeletal tumors. *Right-hand panels:* immunostaining of CD31-positive blood vessels (arrows) within skeletal tumors from animals treated with ZOL or ZOL + daily US. (**b**) Quantification of tumor-cell proliferation, as judged by the percentage of Ki-67–positive nuclei. *Right-hand panels:* Ki-67 nuclear antigen immunostaining within skeletal tumors from animals treated with ZOL or ZOL + daily US. Proliferative cells are stained brown. (**c**) Immunodetection of unprenylated and total Rap1A in protein extracts from skeletal tumors of animals treated with the vehicle, ZOL or ZOL+US. Cropped gels are presented. Full-length blots are presented in Supplementary Figure 6. Tubulin was used as a control for equal protein loading. All data were obtained from different mice on day 32 after tumor cell inoculation. The images shown are examples that best illustrate the effects of the different treatments on bone metastasis formation.

To determine how ZOL+US could decrease skeletal tumor burden, we measured the extent of vascularization and tumor cell proliferation in hind limbs with metastases from mice treated with ZOL, alone or in combination with US. Immunohistochemical analysis of bone metastases with an anti-CD31 antibody that specifically recognizes murine endothelial cells showed that a single treatment with ZOL or a daily treatment with US did not inhibit tumor-associated angiogenesis, compared with vehicle (Fig. 3a). Similarly, ZOL or US treatment of metastatic animals did not affect tumor cell proliferation, as judged by Ki67 nuclear antigen staining of metastatic hind limbs (Fig. 3b). By contrast, ZOL in combination with US statistically significantly decreased both the vascularization and tumor cell proliferation by 70%, compared with ZOL alone (Fig. 3a,b).

To further demonstrate that US treatment was triggering the antitumor activity of ZOL, we measured unprenylated Rap1A levels in bone marrow protein extracts from metastatic hind limbs of animals treated with the vehicle or ZOL (alone or in combination with US). As shown by Western blotting (Fig. 3c), only ZOL+US induced accumulation of unprenylated Rap1A in metastatic bone marrow. This accumulation of the unprenylated form of Rap1A was therefore indicative of the cellular uptake of ZOL and subsequent inhibition of FPP synthase activity.

### Low-intensity ultrasound (US) promotes the release of bisphosphonates from bone mineral

**Figure 4:**
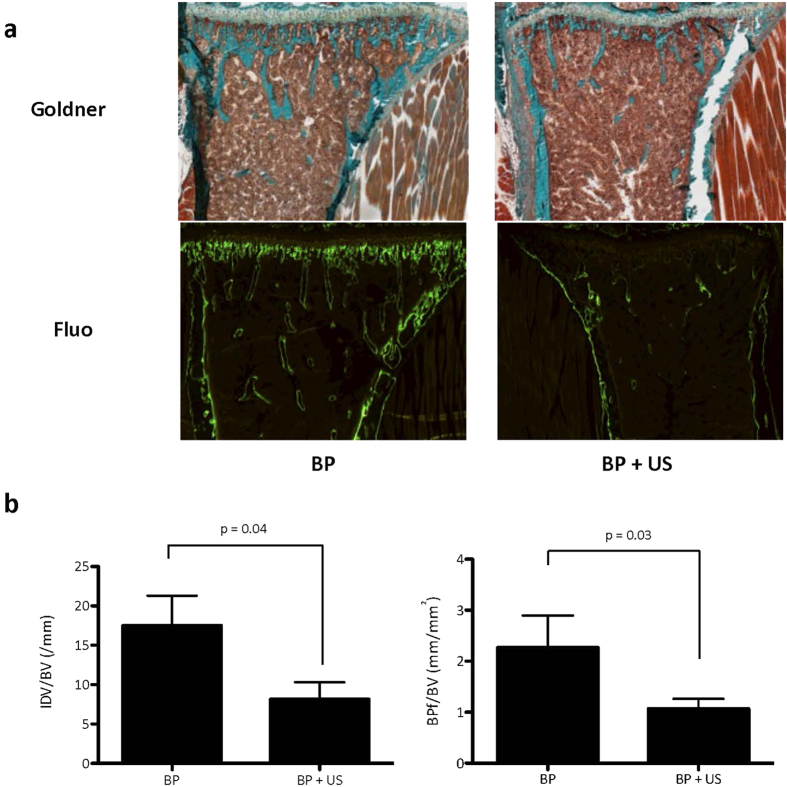
Effects of continuous ultrasound on the binding of fluorescent bisphosphonate FAM-RIS to bone *in vivo*. FAM-RIS (BP) was injected into the tail vein of mice (0.35 mg/kg). Mice were then treated or not treated with low intensity continuous ultrasound (C-US) for 2 weeks. (**a**) Representative images of Goldner trichrome stained tibial tissue sections and corresponding bone tissue sections examined by fluorescence using episcopic light. (**c**) Quantification of the binding of FAM-RIS. Both intensity of fluorescence and bisphosphonate-binding area were quantified. All results are presented as the ratio between fluorescence or bisphosphonate binding area and the bone volume. *Left graph*: Intensity of fluorescent bisphosphonate over bone volume ratio (IDV/BV). *Right graph:* Fluorescent bisphosphonate binding-area over bone volume ratio (BPf/BV).

To understand how US could trigger ZOL antitumor effects, we measured the binding of bisphosphonates by fluorescence microscopy in tibias from animals treated with fluorescently labeled risedronate (FAM-RIS), alone or in combination with US. As shown in Fig. 4, there was a 55% decrease of the fluorescence intensity in tibias from animals treated with FAM-RIS + US (*P* < 0.04), compared with animals treated with FAM-RIS alone. Thus, US promoted the release of bisphosphonates from bone mineral, thereby explaining why there was an accumulation of unprenylated Rap1A in the bone marrow from metastatic animals treated with ZOL+US (Fig. 4).

### Low-intensity ultrasound (US) promotes the antitumor effect of zoledronate (ZOL) in animals bearing subcutaneous breast tumors

**Figure 5:**
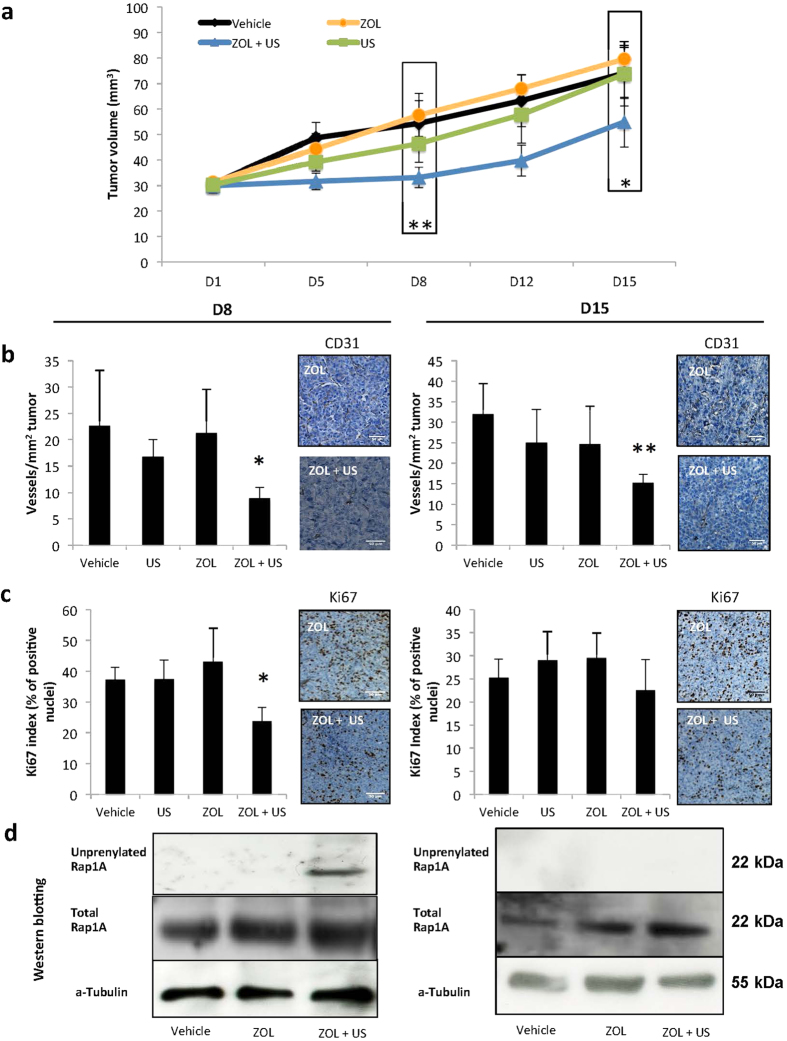
Effects of zoledronate (ZOL), alone or in combination with low-intensity continuous ultrasound (US), on the growth of established subcutaneous breast tumors. (**a**) B02 breast cancer cells were implanted subcutaneously into immunodeficient mice. After subcutaneous tumors reached a volume of 30 mm^3^, mice were treated with the vehicle, ZOL, US or ZOL+US (day 1). Tumor progression was monitored from day 1 (D1) to day 15 (D15), following measurement of the tumor volume with a Vernier caliper. (**b**) Quantification of CD31-positive blood vessels within subcutaneous tumors at D8 and D15. *Upper panels:* immunostaining of CD31-positive blood vessels within tumors from animals treated with ZOL or ZOL + daily US. (**c**) Quantification of Ki-67–positive tumor cells within subcutaneous tumors at D8 and D15. *Upper panels:* Ki-67 nuclear antigen immunostaining within subcutaneous tumors from animals treated with ZOL or ZOL + daily US. (**d**) Immunodetection of unprenylated Rap1A in protein extracts from subcutaneous tumors at D8 and D15. Cropped gels are presented. Full-length blots are presented in Supplementary Figure 8. Tubulin was used as a control for equal protein loading. *, **P < .05 and .002, respectively.

There is evidence that primary breast tumors recruit bone marrow-derived endothelial cell progenitors that then differentiate into mature endothelial cells and contribute to the vascularization of these tumors^24^. We therefore reasoned that, by promoting the release of bisphosphonates from bone mineral, US might assist ZOL in interfering with the vascularization of breast tumors. To address this question, immunodeficient mice bearing subcutaneous B02 breast tumor xenografts were treated with a single dose of ZOL (100 μg/kg body weight), alone or in combination with US (daily). Alternatively, tumor-bearing mice received a daily application of US only. On the first week of treatment (D8), ZOL or US alone did not inhibit B02 tumor growth (Fig. 5a). By contrast, a 42% reduction of the volume of B02 tumors (P < 0.002) was observed in ZOL+US treated mice, compared with vehicle-treated animals (Fig. 5a). In addition, compared with ZOL-treated animals, the weight of tumors from animals treated with ZOL+US was reduced by 34% at D8 (Fig. S7). The smaller size and weight of tumors from ZOL+US-treated animals was associated with decreased tumor-associated angiogenesis, as judged by CD31 immunostaining (Fig. 5b). *In situ* immunodetection of the proliferation marker Ki-67 nuclear antigen in tumors from mice treated with ZOL+US showed also a substantial reduction in proliferative index, compared with tumors from vehicle-treated mice (Fig. 5c).

On the second week of treatment (D15), the outgrowth of subcutaneous tumor xenografts in ZOL+US treated animals was still statistically significantly lower compared with that of vehicle-treated animals (Fig. 5a). There was also a significant reduction of tumor-associated angiogenesis (Fig. 5b). However, this combined treatment did not anymore inhibit the proliferative index of B02 subcutaneous tumors (Fig. 5c). In addition, tumor weights between experimental groups did not anymore differ at D15 (Fig. S4). Thus, the antitumor effect of ZOL that was observed after a one-week treatment of animals with ZOL+US did not persist after two weeks of treatment. Given that a single dose of ZOL was administered to tumor-bearing animals, our results suggested that circulating levels of ZOL liberated from bone mineral gradually decreased over time, ZOL concentrations being too low at D15 to be effective on tumor growth inhibition. Importantly, this contention was supported by the observation that ZOL+US induced accumulation of unprenylated Rap1A in tumor extracts at D8, contrary to D15 (Fig. 5d).

## Discussion

Our results show that a single clinically relevant dose of ZOL produced meaningful antitumor effects in animal models of primary breast tumor and secondary bone metastasis, only when tumor-bearing mice were co-treated with ultrasound (US). In agreement with previous findings^24^, mice with established bone metastases that were treated with a single clinical dose of ZOL had less bone destruction and less skeletal tumor burden, than vehicle-treated animals. This reduced skeletal tumor burden was likely due to the antiresorptive activity of ZOL. Indeed, the skeleton is a rich source of growth factors including transforming growth factor-beta (TGF-β) and insulin-like growth factor (IGF) that are released during bone resorption^1^. By inhibiting bone resorption, bisphosphonates deprive tumor cells of these bone-derived factors that are required for tumor growth^2^,^3^,^6^,^23^,^24^. However, if bisphosphonate treatment decreased skeletal tumor burden solely by reducing bone loss, we would not have expected the treatment combining ZOL and US to have inhibited skeletal tumor burden more than what we observed with ZOL alone. Thus, additional inhibitory mechanisms have happened when using a combined treatment with ZOL and US. In this respect, accumulation of unprenylated Rap1A was detected in skeletal tumors from animals treated with ZOL and US. This accumulation was indicative of the cellular uptake of ZOL and subsequent inhibition of FPP synthase activity, demonstrating that US promoted ZOL penetration within tumors. In addition our results about pure hyperthermia experiments are also in agreement with the fact that hyperthermia increases cell membrane permeability and therefore drug uptake^11^,^12^. Importantly, IPP accumulation and Western Blot analysis demonstrated that US inducing both mechanical and thermal effects were more efficient to promote ZOL penetration into tumor cells than hyperthermia alone. US create heat and mechanical stress, including radiation force effects, acoustic streaming, standing waves and Lamb wave^25^. For example, Lamb waves propagate along the surface of solid materials such as bone surfaces^25^. As demonstrated here using fluorescent risedronate, it is therefore likely that some of these mechanical stresses generated by US mechanically forced the release of ZOL from the bone surface, enabling a continuous impregnation of the bone marrow with low iterative bisphosphonate concentrations, which in turn impeded skeletal tumor outgrowth. This contention was supported by the substantially decreased tumor-associated angiogenesis and tumor cell proliferation *in situ* when ZOL+US treatment was applied.

There is evidence that primary breast tumors recruit bone marrow-derived endothelial cell progenitors that then differentiate into mature endothelial cells and contribute to the vascularization of these tumors^24^. Bisphosphonates exhibit anti-angiogenic properties *in vitro* and *in vivo*, and they reduce tumor-associated angiogenesis in animal models of cancer^3^. We therefore reasoned that, by promoting the release of bisphosphonates from bone mineral, US might assist ZOL in interfering with the vascularization of breast tumors. To address this question, we conducted experiments in mice bearing breast tumors outside bone (*i.e*. subcutaneous tumor xenografts) that were treated with the vehicle or ZOL, alone or in combination with US. We found that US in combination with a single clinical dose of ZOL inhibited subcutaneous tumor growth, compared with ZOL alone or the vehicle. Additionally, tumor growth reduction coincided with decreased tumor-associated angiogenesis and decreased tumor cell proliferation. Importantly, there was also an increased accumulation of unprenylated Rap1A in subcutaneous tumors, demonstrating that there was an uptake of ZOL within these tumors. The bone marrow is a reservoir for CD11b+ myelomonocytic cells^3^. Bone marrow-derived CD11b+ myelomonocytic cells infiltrate distant tumors and contribute to their vascularization by producing matrix metalloprotease-9, which in turn, promotes the release of VEGF from the extracellular matrix^26^. It has been previously shown that treatment of animals with high cumulative doses of ZOL (0.1 mg/kg, daily for 4 weeks) inhibits the infiltration of CD11b+ myelomonocytic cells within mouse mammary tumors^26^. By inducing the release of ZOL from the bone matrix with US, it is likely that a single dose of this bisphosphonate (0.1 mg/kg) also inhibited the infiltration of proangiogenic CD11b+ myelomonocytic cells within human breast tumors. As discussed above, ZOL might also interfere with the recruitment of bone marrow-derived endothelial progenitors to distant tumors. The net result was that ZOL exhibited a systemic antitumor effect when combined with US by reducing tumor-associated angiogenesis. However, this antitumor effect of ZOL disappeared over time, suggesting that bisphosphonate concentrations released from bone mineral (upon US treatment) gradually decreased, and became too low to be effective on inhibition of tumor growth outside bone.

Our results do, to some extent, echo the findings observed in the clinical use of bisphosphonates as adjuvant therapy in breast cancer. Large phase-III clinical trials have shown that adding ZOL to endocrine therapy or chemotherapy improves disease-free survival of patients with endocrine-responsive early breast cancer in a low estrogen environment (*i.e*., following ovarian suppression therapy or in women with established menopause at diagnosis)^2^. For example, the landmark Austrian Breast and Colorectal Study Group (ABCSG)-12 trial showed that the addition of ZOL to hormone therapy for 3 years reduced the risk of disease progression by 36% in premenopausal women with endocrine-responsive stage I or II breast cancer, who were also receiving goserelin to induce artificial menopause^2^. Interestingly, women in the ABCSG-12 trial who received ZOL maintained improvements in relapse-free survival at 84 months’ follow-up and there was also a significant reduction in the risk of death, while their treatment lasted more than 3 years ago^2^. This “carryover” effect of ZOL in the ABCSG-12 trial may be explained by the pharmacological properties of bisphosphonates. It is sustained by our own experimental data, suggesting that the release of ZOL from the skeleton (either naturally or US-induced) enables continuous impregnation of the bone marrow with low iterative bisphosphonate doses that may impede the retention of cancer cells in the bone marrow and/or interfere with the tumor-growth supportive functions of bone-derived factors and bone marrow-derived myelomonocytic cells and endothelial cell progenitors. Additionally, our present findings suggest that US may be an effective strategy to enhance bisphosphonate penetration through tumor tissue. ZOL treatment concurrent with neo-adjuvant chemotherapy has been reported to improve pathologic complete response in patients with breast cancer, compared with chemotherapy alone^27^. US-induced regional hyperthermia has been shown to increase the benefit of neo-adjuvant chemotherapy in patients with localized soft-tissue sarcoma^11^. It is conceivable that an appropriate focusing of the US beam to breast tumors in patients receiving neoadjuvant chemotherapy and ZOL would enhance pathologic complete response rate. In the current study the observed effect comes in part from mechanical stress on bone cells, potentially surface waves that propagate along the bone surface. This hypothesis also makes the results of the cell culture experiments relevant, since the cells are seeded on a rigid petri dish surface, similarly to the way cells are located within the bone. However, the depth of penetration of the surface wave is on the order of the Lamb wavelength in the bone, which, in case of the mouse bone, can cover the entire bone thickness. In a human bone ultrasound will propagate mostly superficially through cortical bone^3^. Therefore, adjustments of the ultrasound transducer geometry and treatment parameters will be performed in order to reach the trabecular bone and the bone marrow in a human bone.

In conclusion, our results demonstrate the potential of low intensity ultrasound as an effective strategy to force bisphosphonate desorption from bone and its penetration through tumor tissue, enabling bisphosphonate antitumor activity (both in bone and outside bone). Our findings made US a promising modality in oncology to trigger anticancer therapy with bisphosphonates.

## Materials and Methods

### Bisphosphonate, cell culture and animals

Zoledronate [1-hydroxy-2-(1H-imidazole-1-yl)ethylidene-bisphosphonic acid], as disodium salt, was provided by Novartis Pharma AG (Basle, Switzerland). Carboxyfluorescein-risedronate (FAM-RIS) was provided by Dr Hal Ebetino (Procter & Gamble Pharmaceuticals, Inc., Mason, OH, USA). Zoledronate and FAM-RIS were dissolved in PBS (pH 7.4, Invitrogen).

Human breast cancer cell lines MCF-7 and T47-D were obtained from the ATCC (American Type Culture Collection) and used within 6 months. Human B02 breast cancer cells, a subpopulation of the MDA-MB-231 cell line, were prepared as described previously^4^,^5^. The MDA-MB-231 and B02 cell lines were authenticated using short tandem repeat analysis. B02 and T47-D cells were cultured in Dulbecco’s Modified Eagle’s Medium (Invitrogen) supplemented with 10% (v/v) fetal bovine serum (Invitrogen) and 1% (v/v) penicillin/streptomycin (Invitrogen). MCF-7 cells were cultured in EMEM medium (LGS Standards for ATCC) supplemented with 10% (v/v) fetal bovine serum, 1% (v/v) penicillin/streptomycin and 10 μg/mL insulin (Sigma-Aldrich). All cell lines were maintained at 37 °C in a 5% CO2 humidified incubator.

Four-week-old female BALB/c athymic (nu/nu) mice were purchased from Janvier (St Berthevin, France). Animals were maintained in a 12-h light-dark cycle and given free access to food and water. All procedures involving animals, including the method by which they were culled and experimental protocols were conducted in accordance with a code of practice established by the ethical committee of the University of Lyon.

### Ultrasound device

Sonication was generated with a flat, piezocomposite air-backed transducer of 40 mm in diameter and with a resonance frequency of 2.9 MHz (Imasonic, Voray-sur-l’Ognon, France). The transducer was inserted into a sterile polyurethane cover (CIV-Flex Transducer cover, CIVCO, Kalona, IA) that was filled with degassed water. This cover attenuated the ultrasound pressure by about 2%, when using a frequency of 2.9 MHz. A peristaltic Masterflex pump (L/S model 7518–60, Cole-Parmer Instruments Co., Chicago, IL) was used to maintain a continuous flow (0.3 l/min) of the degassed water at 20 °C, enabling the cooling of the transducer during *in vivo* experiments. The transducer-driving equipment was composed of a power amplifier (Kalmus model 150 CF, Engineering International, Woodinville, WA) driven by an HP 8116A wave generator (Hewlett Packard GmbH, Boblinger, Germany). A NAP wattmeter/reflectometer (Rohde & Schwarz, Munich, Germany) fitted with its NAP-Z7 probe (Rohde & Schwarz, Munich, Germany) was used to measure forward and reverse electrical power. The wattmeter and the wave generator were connected to a laptop using a GPIB connection.

### Measurement of hyperthermia induced by low-intensity continuous ultrasound

Sonication was delivered in a continuous mode. The duration of each sonication was 30 minutes. Heat created in tissues by ultrasound is directly linked to the acoustic energy, heat diffusion in tissues and perfusion^28^. Adequate acoustic parameters are a trade-off between temperature increase due to the absorption of acoustic energy and temperature decrease due to perfusion and heat diffusion. Therefore, an acoustic power of 12.8 watts was firstly delivered for 7 minutes in order to slightly increase the temperature at the bone-tissue interface up to 43 °C. Then the acoustic power is modulated as a function of time (10.2 W for 2 minutes, 8.2 W for 4 minutes, 8.6 W for 2 minutes and 30 seconds and 8.2 W for the remaining 14 minutes and 30 seconds) to maintain hyperthermia (Fig S2A and S2D). These settings were determined from previous numerical simulations using an in-house software^29^,^30^ as well as *in vitro* and *in vivo* experiments described below. The acoustic-power output and the maximal pressure amplitude as a function of the applied radiofrequency power were measured as described previously^31^. Under these experimental conditions, the maximal peak negative-pressure amplitude calibrated in water was 0.29 MPa. These settings were defined to avoid cavitation while creating hyperthermia and direct mechanical forces.

Previous to therapeutic experiments, these exposure conditions were tested *in vivo* in three mice to demonstrate that creating and maintaining mild HT in hind limbs will be feasible in real conditions without creating any complications or secondary lesions. For this purpose, mice were previously anesthetized with isoflurane gas (4%) and maintained in that condition for the length experiment (isoflurane 1%, oxygen 100 mL/min). Two thin thermocouples (0.33 mm in diameter, MT-29, Physitemp, NJ, USA) were used. One was placed in hind limbs at the bone tissue interface and the other one in the abdominal cavity to check temperature increase produced with these parameters. This study was performed as part of different experiments for other purposes. Animal experiments were performed under an approved research protocol. These experiments conformed to the requirements of the local office of animal experimentation were in accordance with the legal conditions of the French National Commission on Animal (N314, BH2012-06, BH2010-45).

In soft tissues, the force is applied in the direction of wave propagation and the magnitude of the force can be approximated by:


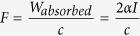


where F [dyn/(1000 cm)^3^ or kg/(s^2^cm^2^)] is the acoustic radiation force, *W*_*absorbed*_ [W/(100 cm)^3^] is the power absorbed by the medium at a given spatial location, c (m/s) is the speed of sound in the medium, α (Np/m) is the absorption coefficient of the medium and I (W/cm^2^) is the temporal average intensity at a given spatial location. The spatial distribution of the radiation force field is, thus, determined by both the transmitted acoustic parameters and the tissue properties.

For the sonication of cultured tumor cells, Petri dishes were placed on an ultrasound absorber (RTV 143, Rhone Poulenc, Milano, Italy) that was previously coated with ultrasound coupling gel to prevent reflection of ultrasound. The transducer and thermocouple were then dipped directly into the cell culture medium. Temperature of each thermocouple was registered every minute. Cavitation was measured using an hydrophone (ONDA HNR-1000, Sunnyvale, CA) placed near the ultrasound transducer pointing towards the exposed medium.

For *in vivo* experiments, the transducer was positioned directly into contact with mice after applying acoustic gel (Ablasonic®, EDAP, Vaux-en-Velin, France) to avoid air between the transducer and the skin. Anesthetized mice were placed in dorsal decubitus position on an ultrasound absorber. For hyperthermia measurement, thermocouples were implanted in the abdominal cavity and the hind limb muscles of mice. Temperature of each thermocouple was registered every thirty seconds. Cavitation was measured using the same methodology as aforementioned.

### Detection of fluorescent bisphosphonate risedronate (FAM-RIS) in hind limbs

Five-week-old mice were injected intravenously with 0.35 mg/kg FAM-RIS or 0.1 ml PBS (vehicle)^32^. For measurement of fluorescence background in bones, control mice received the vehicle only. Mice were then followed until day 13 or treated with US everyday, during 13 days. Mice were culled and hind limbs were collected and fixed in 70% ethanol, then embedded in methylmethacrylate. Undecalcified sections of long bones of 7-μm-thick were cut using a heavy duty microtome (Leica SM2500, Germany) equipped with tungsten carbide knife and stained with Goldner’s trichrome. Images were taken with episcopic fluorescence light using Zeiss Axio Imager (Iena, Germany) equipped with Filter Set 09 (excitation BP 450–490, beamsplitter FT 510, emission LP 515). Intensity of fluorescence and the area of fluorescent bisphosphonate were chosen as two parameters to assess bisphosphonate release related to ultrasonic treatment and measured by ImageJ software. Bone volume was also measured using ImageJ software. Results are presented as a ratio of intensity on bone volume and bisphosphonate volume on bone volume. Experiments were carried out in accordance with the approved guidelines and approved by the ethical committee of the University of Lyon (n°DR2014-12).

### Ultrasound device and measurement of hyperthermia induced by low-intensity continuous ultrasound

Sonication was generated with a flat, piezocomposite air-backed transducer of 40 mm in diameter and with a resonance frequency of 2.9 MHz (Imasonic, Voray-sur-l’Ognon, France). Sonication was delivered in a continuous mode. The duration of each sonication was 30 minutes. The acoustic-power output and the maximal pressure amplitude as a function of the applied radiofrequency power were measured as described previously^33^,^34^. See Supplementary Materials and Methods for further details regarding the *in-vitro* and *in-vivo* experimental conditions for sonication and measurement of hyperthermia induced by low-intensity continuous ultrasound.

### Mouse model of breast cancer bone metastasis

The bone metastasis experiments in mice were conducted as previously described using B02 breast cancer cells^23^,^24^. In this model, mice usually develop bone metastases 18 days after tumor cell injection, as judged by radiography. Osteolytic lesions were identified on radiographs as demarcated radiolucent lesions in the bone. The area of osteolytic lesions was measured and the extent of bone destruction per mouse was expressed in square millimeters. Mice were analyzed by radiography on day 18, and tumor-bearing animals were distributed among the different treatment groups (n = 5–9 mice per group) Anesthetized mice were euthanized by cervical dislocation after radiography on day 32. Experiments were carried out in accordance with the approved guidelines and approved by the ethical committee of the University of Lyon (n BH2010-45).

### Mouse model of subcutaneous breast tumor outgrowth

Subcutaneous tumor xenograft experiments in mice were conducted using B02 breast cancer cells as previously described^4^,^5^. When B02 tumors had reached a volume of 30 mm^3^, mice were randomly assigned to the different treatment groups (n = 7–8 mice/group). Tumor size was calculated by external measurement of the width (m_1_) and length (m_2_) of subcutaneous tumors using a Vernier caliper. Tumor volume (TV) was calculated using the equation TV = (m_1_^2^ × m_2_)/2. Experiments were carried out in accordance with the approved guidelines and approved by the ethical committee of the University of Lyon (n°BH2012-06).

### Treatment protocols for zoledronate (ZOL), mild hyperthermia (HT) and low-intensity ultrasound (US) in cultured tumor cells and tumor-bearing mice

T47D, MCF-7, and B02 breast cancer cells were seeded in 10-cm Petri dishes at 2 × 10^6^ cells/dish overnight at 37 °C in a 5% CO_2_ incubator. Cells were treated with 25-μM ZOL for 1h, then incubated without drug for 23 hours. Alternatively, cells in culture received a continuous treatment with ZOL for 24 hours. For ultrasound experiments, tumor cells previously treated with ZOL or left untreated were sonicated at room temperature for 30 minutes. US-treated cells were then incubated at 37 °C for the rest of the experiment. For hyperthermia experiments, MDA-MB-231/B02 cells were seeded into flasks at 1 million cells/flask. Flasks were immersed during 30 minutes into a warm bath (43 °C) and then incubated for the rest of the experiment. Hyperthermia was generated only *in vitro.* It would have been difficult to induce pure hyperthermia only for small and deep areas in bone with a non-invasive technique that could have been properly compared with ultrasound conditions.

Animals that had developed bone metastases were treated with a single dose of ZOL by subcutaneous injection in 100 μL PBS (vehicle) on day 18 after tumor cell inoculation. ZOL treatment was also administered to animals bearing subcutaneous tumors when these tumors reached a volume of 30 mm^3^. Based on an average body weight of 20 g for 4-week-old mice, ZOL was administered to tumor-bearing mice at a dosage of 100 μg/kg, which was calculated equivalent to the 4-mg clinical dose^24^. Additionally, tumor-bearing mice that were treated with ZOL or the vehicle received a single or a daily 30-min treatment with US. Control tumor-bearing mice received a treatment with vehicle only. All of the mice were sacrificed 2 weeks after the beginning of the treatment.

### Detection of isopentenyl pyrophosphate (IPP)

Bisphosphonate-induced IPP production was measured in human breast cancer cells, as previously described^4^,^5^. ZOL is an inhibitor of farnesyl pyrosphosphate synthase (FPPS) in the signaling mevalonate pathway. FPPS inhibition leads to the accumulation of IPP. Bisphosphonate-induced IPP production was measured in MCF-7 breast cancer cells, as previously described^35^. IPP is thus a surrogate marker for ZOL penetration and action on the mevalonate pathway^36^. Following bisphosphonate treatment, alone or in combination with ultrasound, tumor cells were harvested by scraping, washed in PBS and extracted using ice-cold acetonitrile (300 μL) and water (200 μL). Samples were then concentrated using a Savant Speed Vacuum concentrator and reconstituted within 100 μl of hexylalmine formate buffer containing 0.25-mM NaF and Na3VO4 (to prevent IPP degradation) and 1 μM methyleneadenosine 5′-triphosphate (AppCp), as internal standard. IPP in cell extracts was then quantified by high-performance liquid chromatography negative ion electrospray ionization mass spectrometry (Waters Micromass® ZQ™ Single Quadrupole). IPP calibrator and AppCp internal standard were purchased from Sigma (St. Louis, MO, USA). Quantification of proteins was made using Bradford method and Bradford reagent (Sigma-Aldrich).

### Detection of unprenylated Rap1A and total Rap1A protein

In order to determine the effects of ZOL, US and lovastatin on the intracellular accumulation of unprenylated Rap1A protein, tumor cells in culture were lysed in 200 μL RIPA buffer (Sigma-Aldrich) containing a cocktail of protease inhibitors (AEBSF 104 mM, aprotinin 80 μM, bestatin 4 mM, E-64 1.4 mM, leupeptin 2 mM, pepstatin A 1.5 mM, dilution: 1/100, Sigma). For bone metastasis, the bone marrow of metastatic hind limbs was flushed with culture medium, bone marrow cells were then pelleted by centrifugation and cell pellets were lysed in RIPA buffer. Subcutaneous tumors were minced with a scalpel, homogenized with a homogenizer (Polytron PT 6100) and samples were lysed in RIPA buffer. Lysates (50-μg protein/sample) from bone metastasis and subcutaneous tumors were then electrophoresed under reducing conditions on 4-12% SDS-polyacrylamide gels (Life Technologies) and electrophoresed proteins were then transferred onto polyvinylidene difluoride (PVDF) membranes (Merck Millipore). Membranes were first incubated for 1 hour with 5% (w/v) skimmed milk in TBS buffer containing 0.1% (v/v) Tween 20 to block nonspecific antibody binding. These membranes were then incubated overnight at 4 °C with a goat-polyclonal anti-Rap1A antibody (SantaCruz Biotechnology) targeting the unprenylated form of the small GTPase Rap1A^21^, or an anti-mouse anti-Rap1A monoclonal antibody (SantaCruz Biotechnology), or a α-tubulin mouse monoclonal antibody (Sigma) diluted, respectively, to 1/500 and 1/2,000 in TBS-0.1% (v/v) Tween 20 containing 5% (w/v) skimmed milk. After incubation with primary antibodies, membranes were washed then incubated with horseradish peroxide (HRP)-conjugated donkey anti-goat, (HRP)-conjugated goat anti-mouse (SantaCruz Biotechnology, 1/2,000 dilution) and anti-mouse secondary antibodies (Amersham; 1/2,000 dilution), and immunostaining was performed with enhanced chemiluminescence (ECL) detection system (Perkin Elmer LAS Inc.)^21^. All western blots were realized using samples deriving from a same experiment and all were performed in the same conditions.

### Cell viability assay

Following treatment with ZOL and/or US, tumor cells were further cultured for 24 hours at 37 °C in a 5% C02 incubator, at which time cells were harvested by trypsinization and viable cells were counted under a microscope using the trypan blue exclusion method.

### Bone histology, histomorphometry and tartrate-resistant acid phosphatase (TRAP) staining

Bone histology, histomorphometric analysis and TRAP straining of bone tissue sections were performed as previously described^23^,^24^. Histomorphometric measurements (i.e., bone volume to tissue volume [BV/TV] and tumor burden to soft tissue volume [TB/STV] ratios) were performed in a standard zone of the tibial metaphysis, situated at 0.5 mm from the growth plate, including cortical and trabecular bones. The BV/TV ratio represents the percentage of bone tissue. The TB/STV ratio represents the percentage of tumor tissue. The *in situ* detection of osteoclasts was performed on TRAP-stained longitudinal paraffin-embedded medial sections of tibial metaphysis with the use of a commercial kit (Sigma). Osteoclast resorption surface was calculated as the ratio of TRAP-positive trabecular bone surface to the total trabecular bone surface at the tumor-bone interface.

### Immunohistochemistry

Immunohistochemistry was performed on an automated immunostainer (Ventana Discovery XT, Roche, Meylan, France) using DABmap Kit according to the manufacturer’s instructions. Staining was visualized with DAB solution with 3,3′-diaminobenzidine as a chromogenic substrate. The sections were counterstained with Gill’s hematoxylin. 4-μm tissue sections were preincubated with 1% goat serum then incubated with a rabbit polyclonal anti-CD31 antibody (AnaSpec, Fremont). To measure tumor-cell proliferation, tumor sections were incubated with a rabbit polyclonal anti–Ki-67 antibody (MIB-1, Dako, Trappes, France). Image analysis was performed by using a light microscope (Eclipse E400, Nikon France, Champigny, France) equipped with a tri-CDD video camera (Sony, Japan). Tumor microvessel density was quantified, as previously described^4^,^5^. The mitotic index was expressed as the percentage of Ki-67–positive nuclei. Ki67 analysis was performed using Histolab® software (Microvision Instruments, Evry, France).

### Statistical analysis

All data were analyzed using GraphPad Prism (La Jolla, USA). For *in vitro* and *in vivo* data, pairwise comparisons were carried out by performing nonparametric Mann-Whitney U-test and Wilcoxon test, respectively. The significance level was fixed at *P* = 0.05 with a power of 95%. All statistical tests were two-sided.

## Additional Information

**How to cite this article**: Tardoski, S. *et al.* Low-intensity continuous ultrasound triggers effective bisphosphonate anticancer activity in breast cancer. *Sci. Rep.* **5**, 16354; doi: 10.1038/srep16354 (2015).

## Supplementary Material

Supplementary Information

## Figures and Tables

**Table 1:** Effect of different dosing regimens of zoledronic acid, alone or in combination with low-intensity continuous ultrasound, on the progression of established breast cancer bone metastases, as judged by radiography*.

Dosing regimens	Nber of mice	mm^2^/mouse	*P*, compared with vehicle	*P*, compared with ZOL
Vehicle	10	6.5 ± 0.8	—	
C-US (daily)	13	7.4 ± 1.3	0.60	
ZOL	11	3.0 ± 0.4	<0.001	—
ZOL+C-US (single)	6	3.9 ± 0.9	0.03	0.30
ZOL+C-US (daily)	14	1.3 ± 0.2	<0.0001	<0.001

^*^Data are mean values ± SD from two separate experiments. All measurements were made 32 days after tumor cell injection. *P* values are for pairwise comparisons with the vehicle-treated control group or the ZOL-treated group using the nonparametric Wilcoxon test. *ZOL* = zoledronic acid; *C-US* = low-intensity continuous ultrasound; – = not applicable (referent).

**Table 2:** Effect of different dosing regimens of zoledronic acid, alone or in combination with low-intensity continuous ultrasound, on the progression of established breast cancer bone metastases, as judged by bone histomorphometry*.

Dosing regimens	Nber of mice	Bone volume (BV/TV, %)	*P, vs vehicle*	*P, vs ZOL*	Tumor burden (TB/STV, %)	*P*, *vs* vehicle	*P*, *vs* ZOL
Naïve	4	30 ± 1			0		
Vehicle	6	20 ± 3	—		62 ± 8	—	
C-US, daily	5	19 ± 2	0.65		58 ± 6	0.35	
ZOL	6	28 ± 4	0.04	—	46 ± 11	0.05	—
ZOL+C-US, single	4	28 ± 3	0.03	0.93	32 ± 12	0.05	0.26
ZOL+C-US, daily	10	29 ± 3	<0.01	0.43	11 ± 6	<0.01	<0.01

^*^Data are mean values ± SD from one representative experiment out of two. *n* is the number of mice. All measurements were made 32 days after tumor cell injection. *P* values are for pairwise comparisons with the vehicle-treated control group or the ZOL-treated group using the nonparametric Wilcoxon test. *Naive*: histomorphometric values of hind limb from a naive mouse that did not receive any tumor cells or treatment is shown for comparison. *ZOL* = zoledronic acid; *C-US* = low-intensity continuous ultrasound; *BV/TV* = bone volume-to-tissue volume ratio; *TB/STV* = tumor burden-to-soft tissue volume ratio; – = not applicable (referent).
