# Supplementary material for: Low-intensity continuous ultrasound triggers effective bisphosphonate anticancer activity in breast cancer
Source: Sci Rep. 2015 Nov 18;5:16354. doi: 10.1038/srep16354 (PMC4649676; doi:10.1038/srep16354)

**Low-intensity continuous ultrasound triggers effective bisphosphonate anticancer activity in breast cancer**

Sophie Tardoski^a-d^, Jacqueline Ngo^a,c,d^, Evelyne Gineyts^b-d^ , Jean-Paul Roux^b-d^, Philippe Clézardin^b-d,*^, David Melodelima^a,c,d,*^

^a^ Inserm, U1032, Lyon, F-69424, France

^b^ Inserm, U1033, Lyon, F-69372, France

^c^ University of Lyon, Villeurbanne, France

^d^ LabEx DEVweCAN, Lyon, France

*** Corresponding author:**

David Melodelima, INSERM, UMR 1032, LabEx DEVweCAN, 151 Cours Albert Thomas, 69424 Lyon cedex 03, France.

Tel : 33 4 72 68 19 30

E-mail: david.melodelima@inserm.fr

**SUPPLEMENTARY FIGURE LEGENDS**

**Figure S1:** ***(a)*** B02 breast cancer cells were cultured at 37°C (control) or at 42°C with or without 25-µM ZOL for 1h, then cultured at 37°C without drug for 23h. Alternatively, B02 cells in culture at 37 or 42°C received a 24-h ZOL treatment. Intracellular accumulation of IPP (a surrogate marker of ZOL activity) was measured by mass spectrometry. ***(b)*** B02 breast cancer cells were treated with vehicle (PBS; control) or 25-µM ZOL for 1h, then cultured without drug for 23h. US was given for 30 min. Alternatively, B02 cells in culture received a 24-h ZOL treatment, alone or in combination with US (30 min). Intracellular accumulation of IPP was measured by mass spectrometry. ***(c)*** Bands of unprenylated Rap1A and tubulin by Western blotting using protein extracts from B02 cells treated with hyperthermia or US, alone or in combination with ZOL (1- or 24-h treatment). Tubulin was used as a control for equal protein loading. Cropped gels are presented. Full-length blots are presented in Supplementary Figure S2.

**Figure S2:** Full-length gel of B02 breast cancer cells treated with or without 25-µM ZOL for 1h or 24h and with hyperthermia (42°) or in combination with US (30 min). Bands of unprenylated Rap1A ***(a)***, total Rap1A ***(b)*** and tubulin ***(c)*** by Western Blotting are presented. Positive control is MCF-7 cells treated with lovastatin (5 µM).

**Figure S3:** Full-length gel of B02 breast cancer cells treated with or without 25-µM ZOL for 1h or 24h with or without US (30 min). Bands of unprenylated Rap1A ***(a)***, total Rap1A ***(b)***, tubulin ***(c)*** are presented. B02 and MCF-7 breast cancer cells were treated with 5 μM of the HMG-CoA reductase inhibitor lovastatin (LOV). Bands of unprenylated Rap1A ***(d)***, total Rap1A ***(e)*** and tubulin ***(f)*** are presented. Positive control is MCF-7 cells treated with lovastatin (5 µM).

**Figure S4:** Experimental device for ultrasound treatment of mice. ***(a)*** Time-dependent increase of temperature in human B02 breast cancer cells cultured in Petri dishes then treated with ultrasound for 30 min. Thermocouple was placed directly into the cell culture medium. One probe was placed at the rim of the transducer (temperature 1). The second one was placed in the middle of the Petri Dish (temperature 2). ***(b)*** Measurement of acoustic cavitation during the course of the treatment of B02 cells with ultrasound for 30 min. Cavitation was measured using a hydrophone placed near the ultrasound transducer pointing towards the exposed medium. Wideband emission and half-harmonics of the fundamental frequency were considered as a signature for stable and unstable cavitation, respectively. ***(c)*** Schematic representation of the ultrasound device. Anesthetized mice were placed into a dorsal decubitus position. Ultrasound transducer was immersed into acoustic coupling gel. *Right-hand panel*: representative photograph of the transducer. *1*: cooling system, *2*: sterile cover, *3*: ultrasound probe, *4*: anesthetic mask, *5*: anesthetized mouse, *6*: acoustic coupling gel, *7*: absorbant. ***(d)*** Time-dependent increase of the temperature in hind limb muscles of mice that were treated with ultrasound for 30 min. Thermocouples were inserted into hind limb muscles and the abdominal cavity. Temperature was monitored to ensure an increase to 42°C into hind limbs. Data are the mean ± SD of 3 mice. ***(e)*** Measurement of the cavitation during the course of the treatment of mice with ultrasound for 30 min.

**Figure S5:** Effects of the bisphosphonate zoledronic acid (ZOL), alone or in combination with low-intensity continuous ultrasound (US), on proliferation of ***(a)*** B02, ***(b)*** MCF-7 and ***(c)*** T47-D breast cancer cells *in vitro*. Tumor cells were treated with vehicle, 25-µM ZOL for 24h, US for 30 min or a combination of ZOL with C-US. At day 3, tumor cells in culture were detached and counted using the trypan blue exclusion method. Results are expressed as the mean ± SD.

**Figure S6:** Full-length gel of unprenylated ***(a)*** and total Rap1A ***(b)*** in protein extracts from skeletal tumors of animals treated with the vehicle, ZOL or ZOL + US. Tubulin was used as a control for equal protein loading ***(c)***. Positive control is MCF-7 cells treated with lovastatin (5 µM).

**Figure S7:** Weight of subcutaneous breast tumors. B02 breast cancer cells were implanted subcutaneously into immunodeficient mice. After subcutaneous tumors reached a volume of 30 mm^3^, mice were randomized (D1) then treated with the vehicle, ZOL, US or ZOL+US. Tumor progression was monitored from day 1 (D1) to day 15 (D15), following measurement of the tumor volume with a Vernier caliper. ***(a)*** and ***(b)*** two series of animals were culled at D8 and D15, respectively, then tumors were collected and weighted. Results are expressed as the mean ± SD.

**Figure S8:** Full-length gel of unprenylated Rap1A in protein extracts from subcutaneous tumors at D8 ***(a)*** and D15 ***(c)***. Tubulin was used as a control for equal protein loading for D8 ***(b)*** and D15 ***(d)***. Positive control is MCF-7 cells treated with lovastatin (5 µM).


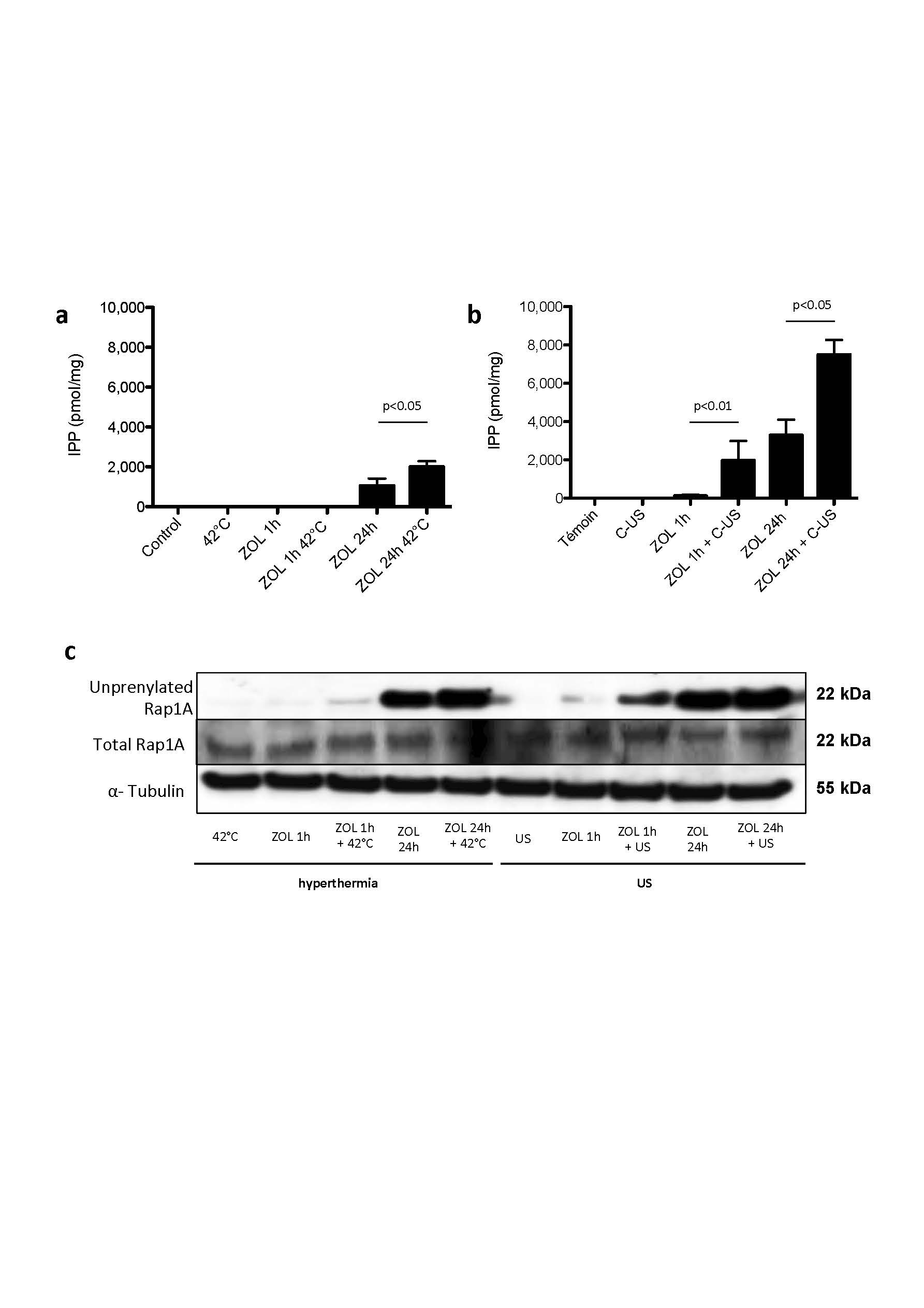


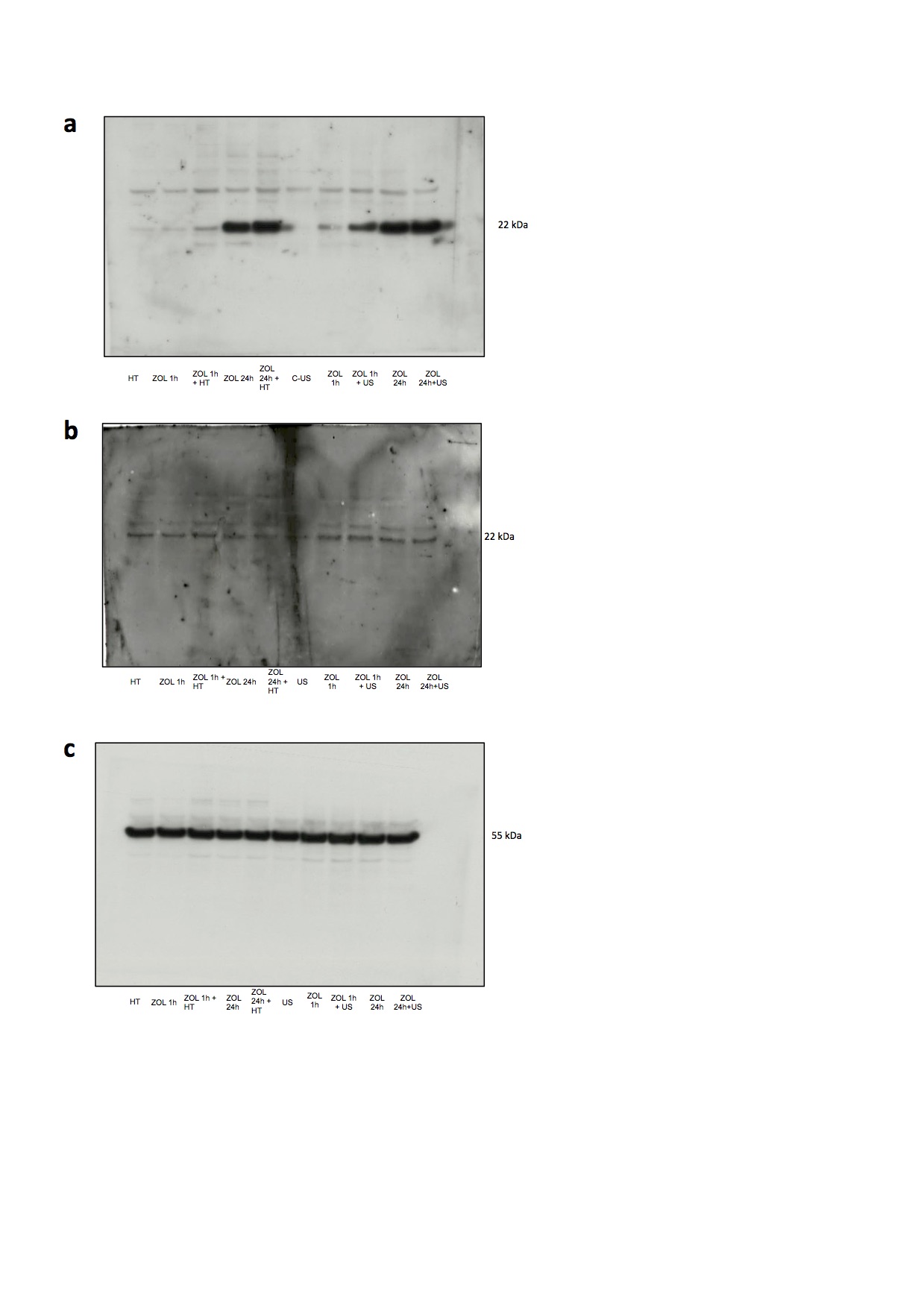


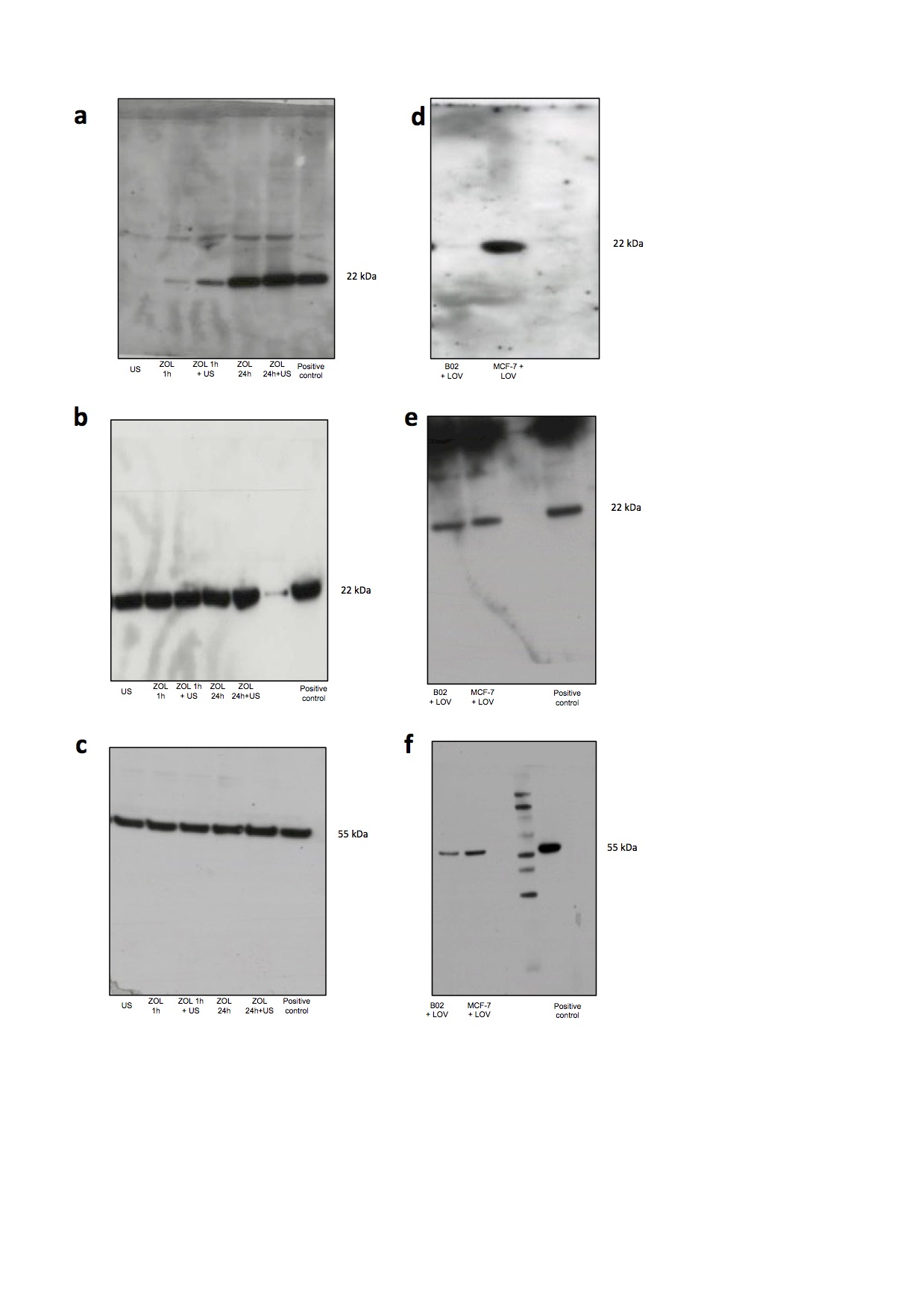


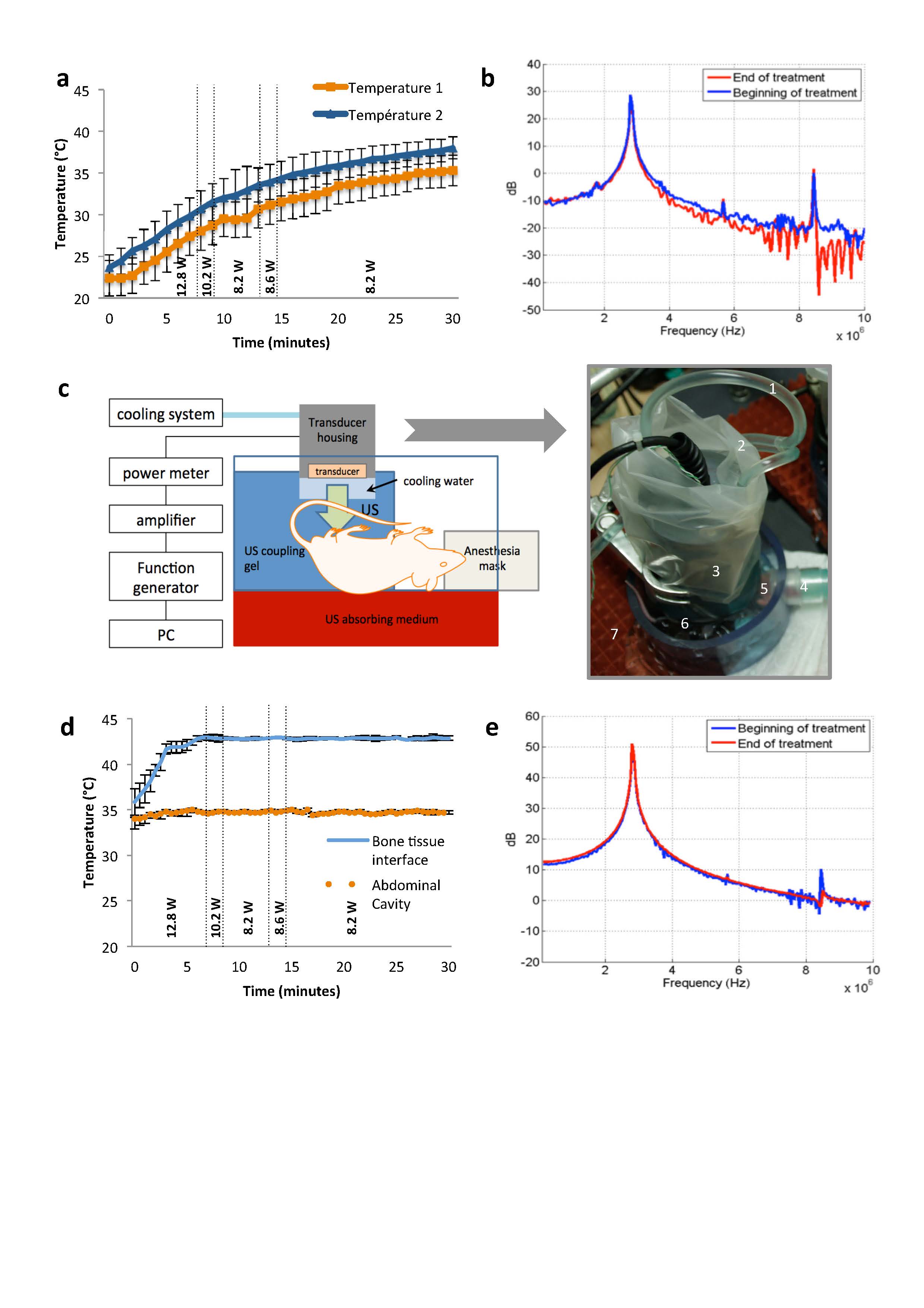


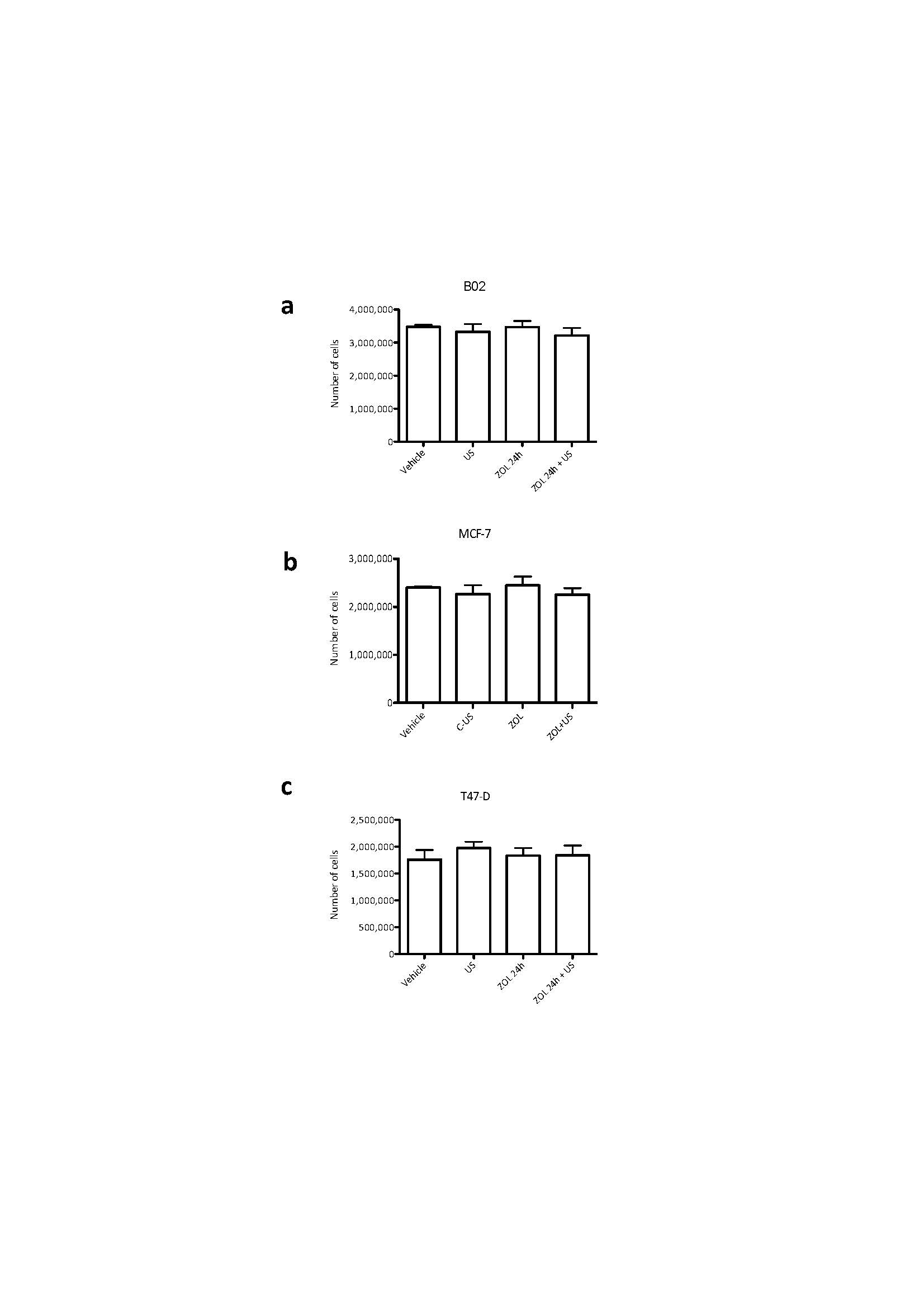


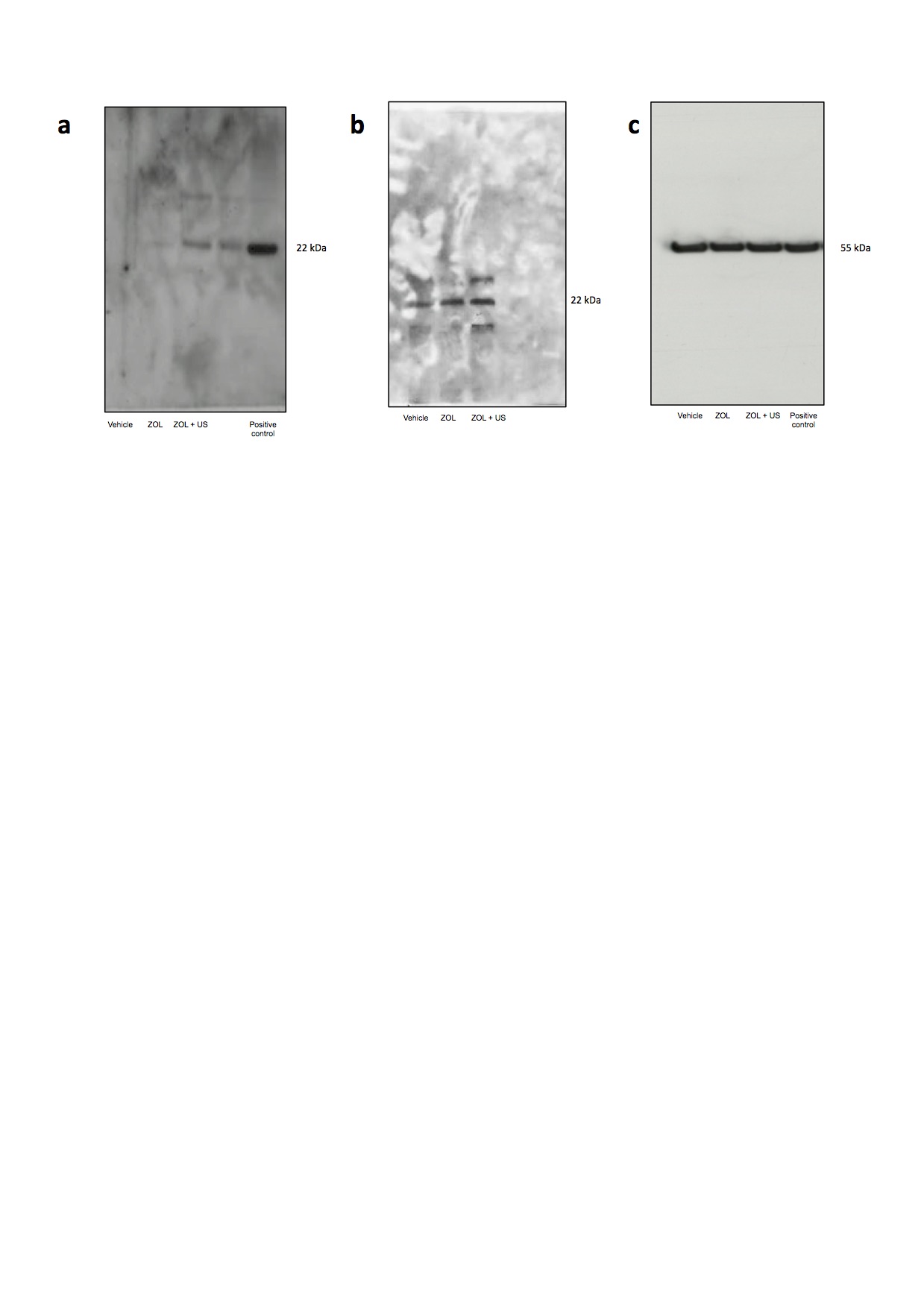


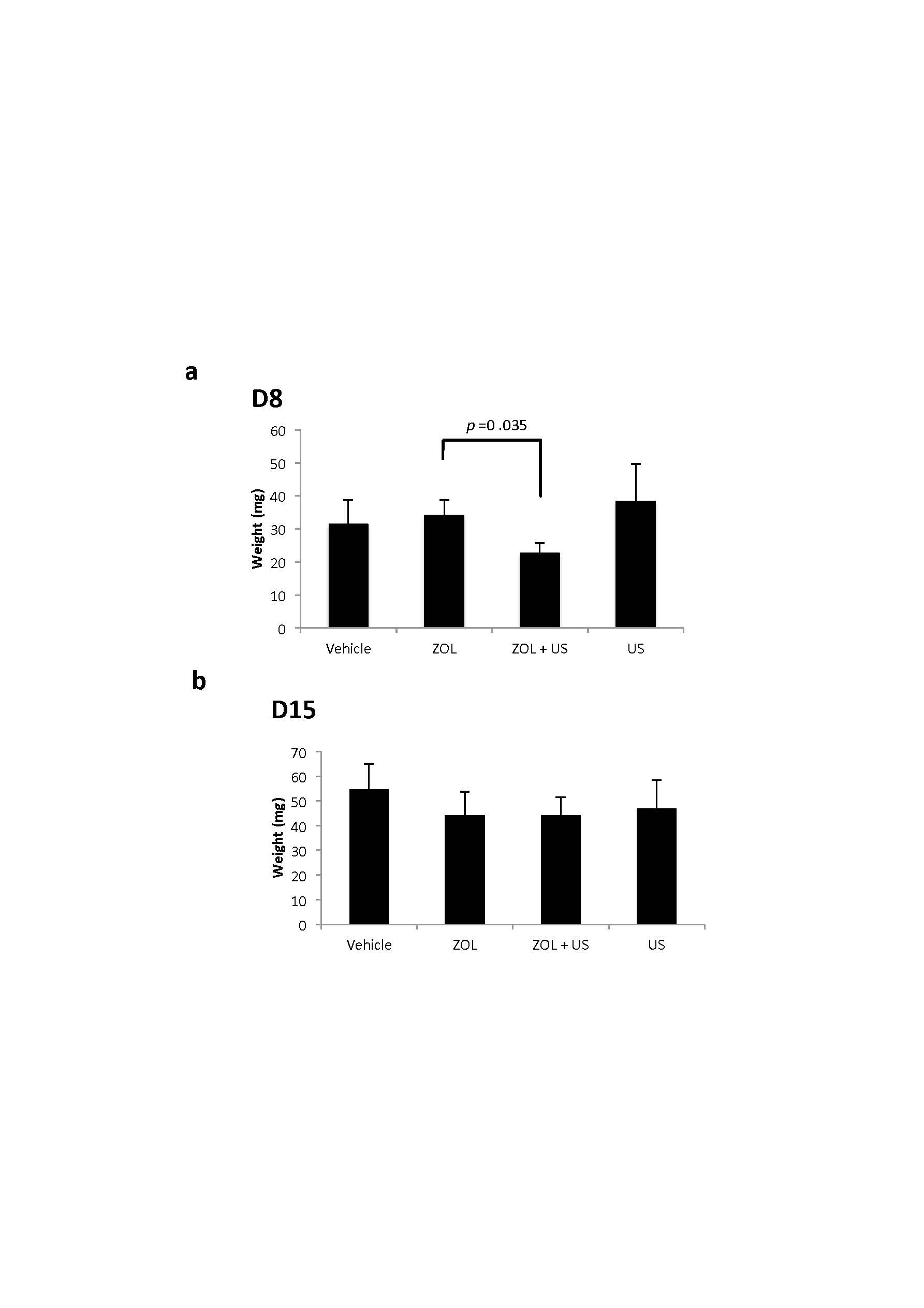

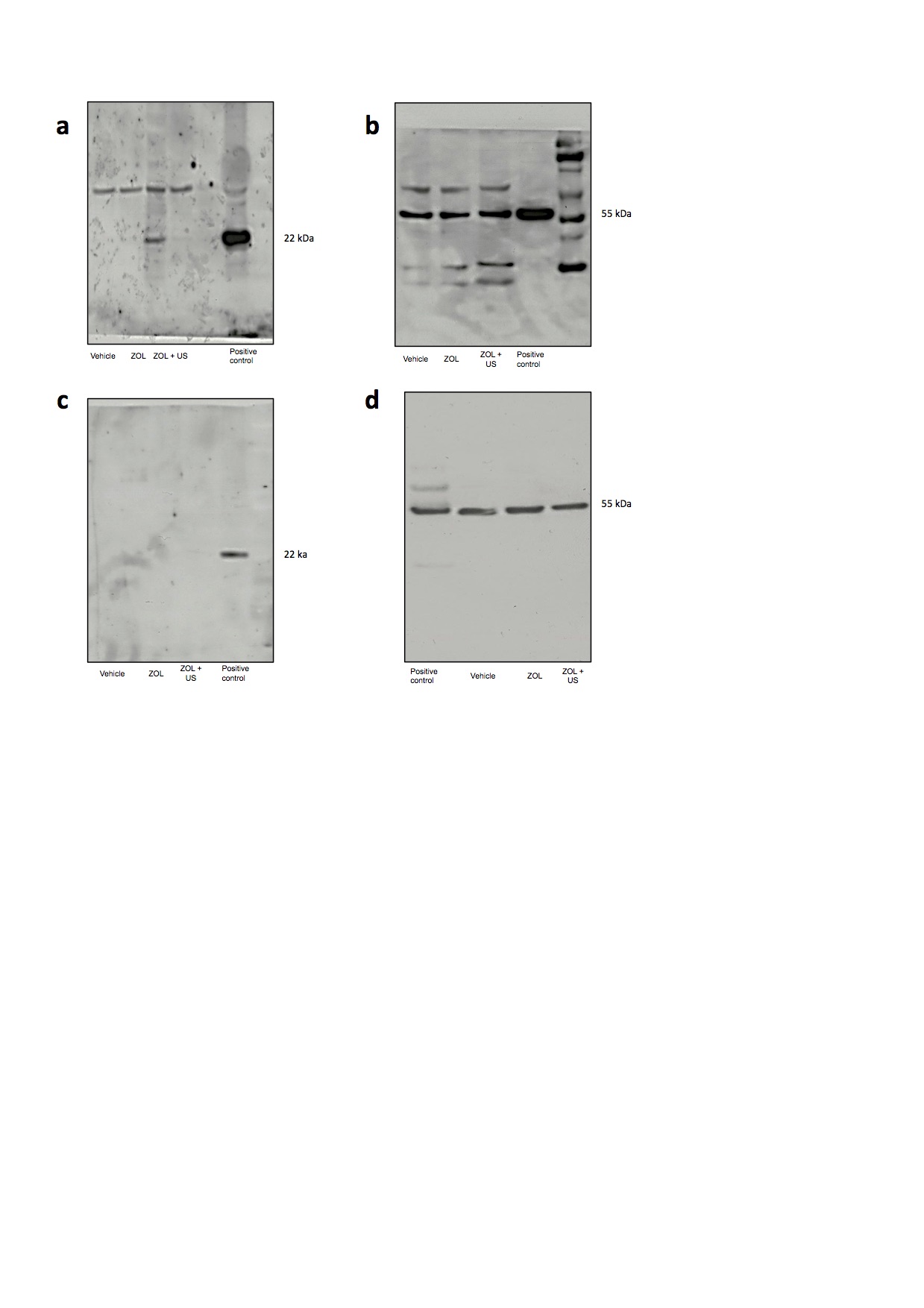

Supplement: Supplementary Information [file srep16354-s1.doc]
